# A structured review of placental morphology and histopathological lesions associated with SARS-CoV-2 infection

**DOI:** 10.1016/j.placenta.2020.08.018

**Published:** 2020-11

**Authors:** Megan C. Sharps, Dexter J.L. Hayes, Stacey Lee, Zhiyong Zou, Chloe A. Brady, Yousef Almoghrabi, Alan Kerby, Kajal K. Tamber, Carolyn J. Jones, Kristina M. Adams Waldorf, Alexander E.P. Heazell

**Affiliations:** a Tommy's Maternal and Fetal Health Research Centre, Faculty of Biology, Medicine & Health, 5th Floor St. Mary's Hospital, University of Manchester, Manchester, UK; b Division of Pharmacy and Optometry, Faculty of Biology, Medicine & Health, University of Manchester, UK; c Department of Obstetrics & Gynecology, University of Washington, Seattle, USA; d St. Mary's Hospital, Manchester University NHS Foundation Trust, Manchester Academic Health Science Centre, Manchester, UK

**Keywords:** SARS-CoV-2, COVID-19, SARS-CoV-1, MERS-CoV, Vertical transmission, Placenta, Pathology

## Abstract

The severe acute respiratory syndrome coronavirus 2 (SARS-CoV-2), a novel coronavirus, was first identified after a cluster of cases in Wuhan, China in December 2019. Whether vertical transmission or placental pathology might occur following maternal infection during pregnancy remains unknown. This review aimed to summarise all studies that examined the placenta or neonates following infection with SARS-CoV-2, or closely related highly pathogenic coronavirus (SARS-CoV-1, or the Middle East respiratory syndrome coronavirus (MERS-CoV)). Structured literature searches found 50 studies that met the inclusion criteria.

Twenty studies reported placental histopathology findings in third trimester placentas following maternal SARS-CoV-2 infection. Using the Amsterdam Consensus criteria to categorise the histopathology results, evidence of both fetal vascular malperfusion (35.3% of cases; 95% Confidence Interval (CI) 27.7–43.0%) and maternal vascular malperfusion (46% of cases; 95% CI 38.0–54.0%) were reported, along with evidence of inflammation in the placentas (villitis 8.7% cases, intervillositis 5.3% of cases, chorioamnionitis 6% of cases). The placental pathologies observed in SARS-CoV-2 were consistent with findings following maternal SARS-CoV-1 infection. Of those tested, a minority of neonates (2%) and placental samples tested positive for SARS-CoV-2 infection (21%).

Limited conclusions can be drawn about the effect of maternal SARS-CoV-2 infection on placental pathology as most lack control groups and the majority of reports followed third trimester infection. Collaboration to maximise the number of samples examined will increase the reliability and generalisability of findings. A better understanding of the association between maternal SARS-CoV-2 infection and placental pathology will inform maternity care during the coronavirus pandemic.

## Highlights

• Placental findings following maternal COVID-19, SARS or MERS were reviewed.
• Minority of infants (2%) and placentas (21%) tested were positive for COVID-19.
• Fetal and maternal vascular malperfusion were most frequently noted following COVID-19.
• Minority of placentas demonstrated inflammatory lesions.
• The association between maternal COVID infection and placental pathology is unclear.

## 1 Introduction

In December 2019, the severe acute respiratory syndrome coronavirus 2 (SARS-CoV-2) was reported in Wuhan, China [1]. SARS-CoV-2 was determined to represent a highly pathogenic coronavirus similar to SARS-CoV-1 and the Middle East Severe Respiratory Syndrome coronavirus (MERS-CoV) [2]. Coronaviruses are positive single stranded RNA viruses [3] and, as SARS-CoV-2 shared homology with a spike protein binding domain with SARS-CoV-1, their pathogenesis was anticipated to be similar [4]. Despite these similarities, an early review found less severe outcomes of COVID-19 (the disease caused by SARS-CoV-2 infection) in pregnancy than SARS and MERS [5]. In 12 pregnancies affected by SARS, there were 3 maternal deaths, 4 first trimester miscarriages, 2 cases of fetal growth restriction (FGR) and 4 preterm births. Eleven cases of MERS during pregnancy resulted in varied adverse clinical outcomes, which included: 1 preterm birth, 3 maternal deaths, 1 stillbirth and 2 neonatal deaths. In the 38 COVID-19 cases there were 9 preterm births, 1 neonatal death and no maternal deaths [5]. A second review examined 49 studies for evidence of transmission of SARS-CoV-2 to the neonate [6]. Neonatal infection was 2.7% in vaginal deliveries and 5.3% in Caesarean sections; this review concluded that breastfeeding, mother-infant contact and the method of delivery did not increase the risk for neonatal SARS-CoV-2 infection. It did not examine the potential of in utero infection.

The largest series to date from the UK reported the incidence of SARS-CoV-2 infection to be 4.9 per 1000 pregnant women (0.49%) [7] which is higher than the overall population (0.27%) in the same time period [8]. Of 427 pregnant women, 40 (9%) required critical care and 5 women died [9]. One quarter of births were preterm, with 46% of those COVID-19 related. Approximately 59% of births were by Caesarean section, of which 27% were due to maternal compromise. During this time, there were 4 pregnancy losses in women admitted to hospital (0.9%).

Although there are an increasing number of studies examining the clinical outcomes of women diagnosed with COVID-19 during pregnancy, and their infants, the reasons underpinning any association with an adverse outcome remain unclear. As the placenta is the maternal-fetal interface, it not only has an essential role in protecting the fetus from infection but can also be affected by an adverse maternal environment. Notably, severe COVID-19 infection results in sustained hypoxia [10], an insult implicated in FGR, pre-eclampsia and stillbirth. Furthermore, vertical transmission of infectious agents can occur either during pregnancy or childbirth. In the former case, infectious agents may cross the placenta leading to fetal abnormalities (e.g. microcephaly in Zika virus infection [11]); alternatively, infectious agents may directly cause placental damage (e.g. villitis from cytomegalovirus [12]) leading to FGR or fetal demise. In either case, evidence of placental transmission or damage is needed to establish a mechanistic link between maternal infection and fetal outcome.

To identify evidence of either of these placental events, we conducted a structured review to identify publications reporting pregnancy outcome in women with COVID-19 and which describe placental findings, in order to evaluate evidence for vertical transmission and/or changes in placental morphology in women with SARS-CoV-2 infection.

## 2 Methods

### 2.1 Literature searches

Systematic literature searches were performed in Medline; an example literature search is provided (Supplementary File 1). Searches were also performed in Google Scholar and Medrxiv using keywords: COVID-19, pregnancy, and placenta. Reference lists of included papers and relevant reviews were also searched. Searches were not limited by year. All studies were screened for inclusion and data were extracted by two review authors, disagreements were resolved by a senior author. The date of the last search was the 23^rd^ July 2020.

### 2.2 Inclusion criteria

Study inclusion required reporting on some aspect of placental morphology or have tested for the virus in the placenta, or established the presence of antibodies in the neonate, in pregnant women who have tested positive for SARS-CoV-2, SARS-CoV-1, or MERS-CoV at any point during their pregnancy. Studies published in any language were considered, as were preprints in openly-accessible archives.

### 2.3 Data extraction

Data were extracted from each study using a standardised tool including; number of cases, maternal and neonatal clinical data, the interval between diagnosis of infection and birth, mode of delivery, gestation at birth, placental testing performed, and the results of any testing. Studies in other languages were translated into English. The results of the histopathological findings were categorised using the Amsterdam Consensus Criteria [13].

## 3 Results

**Fig. 1:**
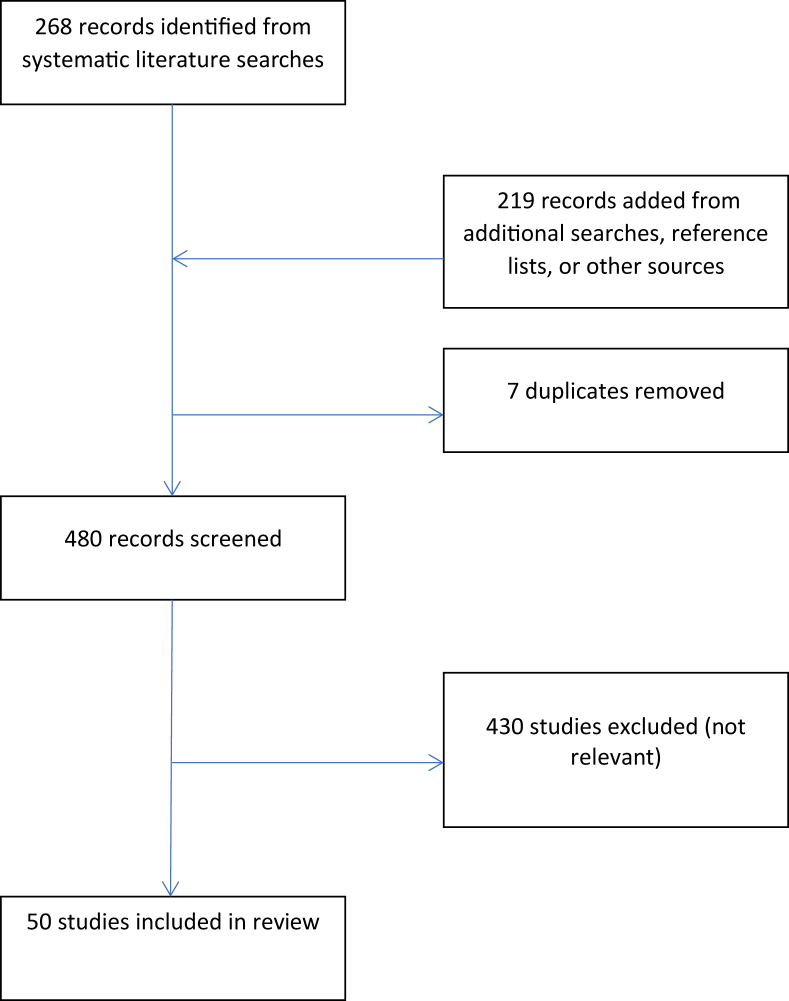
Flow chart of the screening process.

Our search strategy identified 480 studies, 50 of which met the inclusion criteria. A flow diagram illustrating the screening process is shown in Fig. 1. Characteristics of included studies are described in Table 1 and Table 3.

**Table 1:** **Characteristics of SARS-CoV-2 included studies.** * Initially presented as a decimal fraction. Calculated: when gestation at birth was calculated according to timeline of events, VD: vaginal delivery, CS: Caesarean section, H&E: haematoxylin and eosin, IHC: immunohistochemistry, TEM: transmission electron microscopy, PCR: polymerase chain reaction, NS: non-significant, MVM: maternal vascular malperfusion, FVM: fetal vascular malperfusion.

Study	Number of women	Clinical data	Mothers COVID- 19 Positive	Method of delivery and gestation at birth	Investigations	Results
Algarroba GN et al., 2020	1	Live female infant born due to deteriorating maternal condition, birthweight 1340 g, 5 min Apgar score of 5. Neonate tested negative.	Yes (unclear when tested) but came in with diagnosis.	CS 28 + 5 weeks (calculated)	PCR on neonatal samples. Placental histopathology TEM.	Placental histology: mature chorionic villi with focal villous oedema, decidual vasculopathy. Single virion in syncytiotrophoblast, single virion in microvillus, single virion found in core of terminal villus.
Algeri P et al., 2020	5	One case had gestational diabetes, two cases had ruptures of membranes. One case born severely premature. All infants were live born. Twins had low birth weight (<2500 g) and mother given corticosteroids. All neonates tested negative.	Yes, two cases diagnosed after delivery, three cases positive on admission.	4 CS, 1 VD, 27 + 4 to 38 + 4 weeks	One placenta sent to histology. PCR on four neonatal swabs (at birth and after 1 month)	Placental histology showed no signs of infection/inflammation, despite suspected chorioamnionitis during pregnancy. Bacterial swab of placenta was negative.
Baergen RN & Heller DS 2020	20 (1 set of twins)	Two mothers had preeclampsia and 1 had hypertension Two infants (2/10) were small for gestational age. All neonates tested negative.	Yes, all 20 tested positive (some asymptomatic) definitely at birth, 3 probably before.	5 CS, 15 VD 32 + 2 to 40 + 4 weeks	RT-PCR on neonatal samples, placental histopathology.	Placental histology: 9 fetal vascular malperfusion (3 intramural fibrin deposition in 1 or 2 foci, 2 villous stromal-vascular karyorrhexis, 4 multiple lesions), 3 cases with thrombi, 6 cases of meconium macrophages, 5 lesions of maternal vascular malperfusion, focal increase in perivillous fibrin deposition. One case of ascending infection with acute chorioamnionitis and acute funisitis. Four cases of chronic villitis (2 high grade, 1 associated with obliterative vasculopathy). In the twin case, one had a villous infarct and the other had high-grade chronic villitis. Eight cases with no gross umbilical cord abnormality associated with malperfusion.
Baud D et al., 2020	1	Nineteen weeks' gestation presented with contractions and fever. Bulging membranes on vaginal examination on admission. Stillborn infant born vaginally after 10 h of labour. Stillborn, birthweight not reported. Autopsy - no malformation.	Yes, 2 days before birth.	Miscarriage. VD 19 weeks	RT-PCR on amniotic fluid, fetal autopsy and fetal blood. Histology on placenta.	Fetal autopsy showed no malformations, and fetal lung, liver, and thymus biopsies were negative for SAR-CoV-2.Placental histology. Fetal surface was disinfected and sampled, samples tested positive. Histology showed monocytes and neutrophils in subchorial space, increased intervillous fibrin deposition, funisitis present. No bacterial or fungal infection. PCR was negative.
Chen S et al., 2020	3	Case 1: complete placenta previa Case 2: Acute cholecystitis, placental abruption Case 3: Complete placenta previa, scarred uterus. One preterm birth. All neonates tested negative.	Yes, 2 cases were positive (4 days and 2 days after delivery), one negative result but CT scan indicated SARS-CoV-2 infection (1 day after delivery).	3 CS 35–39 weeks	RT-PCR on neonatal swab. H&E of placenta, RT-PCR of full thickness placenta, membranes, and umbilical cord.	Placental histology: Placenta 1: chorionic haemangioma, fibrin in extravillous insterstitium, local end villous syncytial nodules increased. Placenta 2: infarction, increased interstitial and perivillous fibrin deposition with increased syncytial nodules. Placenta 3: interstitial and perivillous fibrin deposition. All three placentas tested negative for COVID-19.
Chen Siyu et al., 2020	5	Two with gestational diabetes, one with preeclampsia. All neonates tested negative. Apgar score of 10. No complications observed in the neonates.	All 5 positive, likely tested at or after birth.	3 VD, 2 CS 38 + 6–40 + 4 weeks.	qRT-PCR on neonatal samples. Placental histology.	Five placentas were histologically normal (no infarctions or chorionic amniotic inflammation).
Chen X et al., 2020	3	Case 1: the pregnancy was terminated 14 days after discharge. Case 2: pregnancy continued. Fetal growth was normal during the treatment but outcome unclear Case 3: emergency CS due to abnormal fetal heart rate but a healthy infant. Neonate tested negative. Methylprednisolone given to cases 2 and 3.	Yes, tested positive. Case 1: 30 days before termination. Case 2: unclear. Case 3:4 days after delivery.	1 third trimester CS, 1 termination of pregnancy, 1 unclear if delivered.	Examination of placenta, amniotic fluid and umbilical cord. PCR testing of placenta, umbilical cord blood, amniotic fluid and neonatal swab.	No abnormalities in the amniotic fluid, umbilical cord or placenta.
Dong L et al., 2020	1	No evidence of pregnancy complications. Corticosteroids administered. Normal birthweight, Apgar score of 10.	Yes, tested positive 3 weeks and 4 days before birth.	CS 37 + 6 weeks (calculated)	Neonatal IgM and IgG, cytokines. RT-PCR on nasopharyngeal swabs.	Neonate tested negative. Had significantly elevated IgM and IgG antibodies at 2 h old, also increased IL-6 and IL-10. IgM and IgG were still elevated at 2 weeks of age.
Fan C et al., 2020	2	No evidence of pregnancy complications. Corticosteroids administered. Normal birthweights, Apgar scores of 10.	Yes, Patient 1: tested positive 7 days before birth. Patient 2: tested positive 4 days before birth.	2 CS 36 + 5, 39 weeks	qRT-PCR on nasopharyngeal swab, cord blood, placenta tissue, amniotic fluid and placenta.	Placenta 1: placenta, cord blood and amniotic fluid tested negative. Placenta 2: “product of conception” was negative.
Ferraiolo A et al., 2020	1	No evidence of pregnancy complications. Apgar scores of 9–10.	Yes, positive after delivery.	CS 38 + 4 weeks	PCR on placental samples, neonatal swab at birth and 24 h. Anti-SARS-CoV-2 serology for IgM and IgG at 10 days old.	Neonate tested negative (first swab inconclusive, 24 h swab negative). 10 day serology tested negative. Placenta tested positive for RNA. Mild subchorionic deposition of fibrin, single ischemic area, focal haemorrhages in amniochorial membranes. Vessels in cord slightly hyperspiralized, no inflammation of membranes or funisitis. Delayed villous maturation. Terminal villi had capillary congestion and focal microchorangiosis. Modest fibrin deposition, villous agglutination, multiple organizing intervillous haemorrhage.
Grimminck K et al., 2020	1	Pre-existing hypertension and stable SLE (on azathioprine). Corticosteroids administered. Birthweight 30th centile (2880 g). Neonate tested negative.	Yes, tested positive on admission.	VD 38 + 2 weeks (calculated)	Maternal and fetal sides of the placenta were sampled. Neonatal oropharyngeal swab.	Placenta tested negative.
Hosier H et al., 2020	1	Severe preeclampsia with placental abruption. Maternal IgM and IgG were highest for any COVID-19 patient in the hospital. Severe hypertension upon admission (previous pregnancy complicated by gestational hypertension at term).	Yes, tested positive on admission.	Termination of pregnancy. 22 weeks	Serology for mother. IHC, *in situ* hybridisation and EM on placenta. PCR on the placenta and umbilical cord, placental histology, PCR on fetal autopsy.	Neonatal samples tested negative. Placental and cord tested positive for COVID-19 RNA. Placental histology: focal placental infarct (indicating abruption), diffuse perivillous fibrin, presence of macrophages and T cells, SARS-CoV-2 spike protein located in the syncytiotrophoblast, EM found viral particles in “cytosol of placental cells”.
Hsu AL et al., 2020	1	No evidence of pregnancy complications. Apgar scores of 8–9. Neonate tested negative.	Yes, 2 days before admission	VD 40 + 4 weeks	Neonatal PCR swab at 24 h, histopathology, IHC for SARS-CoV-2	Placental histology: no gross lesions, decidua had scattered arterioles and thickened smooth muscle (consistent with hypertropic arteriolopathy and subchorionic laminar necrosis). Focal lympho-histiocytic inflammation (consistent with chronic villitis), scattered island of extravillous trophoblasts. IHC found SARS-CoV-2 antigens throughout the placenta, under the umbilical cord, central and peripheral disc in chorionic villi endothelial cells, but rarely in trophoblast.
Huang JW et al., 2020	1	No evidence of pregnancy complications. Fetal distress prompted CS. Neonate alive, no detailed information. Neonate tested negative.	Yes, tested positive; unclear when.	CS 35 + 4 weeks (calculated)	SARS-CoV-2 nucleic acid testing of mother (stool and sputum), neonate (oropharyngeal swab). Cord blood, amniotic fluid, and placenta also tested.	All samples tested negative.
Kalafat E et al., 2020	1	No evidence of pregnancy complications. Single episode of reduced fetal movements. Normal birthweight (2790 g). Apgar score 9 at 5 min. Neonate tested negative.	Yes, tested positive 5 days before birth.	CS 36 + 1 weeks (calculated)	RT-PCR on placenta (“maternal and fetal sides”), cord blood and neonatal swabs.	All samples tested negative.
Kirstman M et al., 2020	1	Familial neutropenia, gestational diabetes, frequent bacterial infections. Apgar scores of 9 Neutropenic and mild hyperthermia so admitted to NICU after 37 h. All neonatal samples tested positive.	Yes, tested positive on admission (1 day before birth).	CS 35 + 5 weeks	Placental swabs of maternal and fetal sides for RT-PCR, histopathology, neonatal nasopharyngeal swab on day of birth, day 2 and day 7. Neonatal plasma on day 4.	Placenta tested positive. Placental histology: multiple areas of inflammatory cell infiltrates (CD68 macrophages, T cells, B cells and neutrophils) in the intervillous space consistent with chronic histiocytic intervillositis but differed: widespread infarction and clustering of inflammatory cells around chorionic villi.
Kuhrt K et al., 2020	1 (set of twins)	Twin pregnancy. Had a placental abruption, 400 ml retroplacental clot, significant intrauterine clots. Given dexamethasone for fetal lung maturation. Normal birthweight (twin one 2190 g, twin two 2160 g). Apgar score 8 and 9 at 5 min. Both twins tested negative.	Yes, tested positive two days before birth.	CS 32 + 6 weeks	PCR on neonatal samples. Placental histology.	Placental histology was clear of leukocyte invasion and fetal inflammatory response, but evidence of accelerated villous maturation, hypoperfusion noted, placental abruption.
Lang GJ et al., 2020	1	No evidence of pregnancy complications. Fetal distress prompted CS. Live birth, male, Apgar 9 and 10 at 1 and 5 min. Neonate tested negative.	Yes, tested positive 4 days before delivery.	CS 35 + 4 weeks (calculated)	RT-PCR on cord blood, amniotic fluid and placenta. Neonatal oropharyngeal swab.	All results were negative.
Lee DH et al., 2020	1	No evidence of pregnancy complications. Normal birthweight (3130 g). Apgar score 10 at 5 min. Neonate tested negative.	Yes, tested positive 1 week 4 days before birth.	CS 37 + 6 weeks	PCR on neonatal samples. RT-PCR on placenta, amniotic fluid and cord blood.	All samples tested negative.
Li Y et al., 2020	1	No evidence of pregnancy complications. Given methylprednisolone.Normal male infant “without complications”. Neonate tested negative.	Yes, tested positive 4 days before birth.	CS 35 + 4 weeks (calculated)	Neonatal oropharyngeal swab tested for RNA. Also tested the placenta, amniotic fluid and cord blood for RNA.	All samples tested negative.
Liu W et al., 2020	3 (1 tested the placenta)	One case had gestational diabetes. Case 1 and 2 received glucocorticoids. Normal birthweights (3250 g, 3250 g, 3670 g). All had Apgar scores of 9 at 5 min. Neonates tested negative.	Yes, all 3 tested positive, 1st patient on day of birth, 2nd patient day before birth, 3rd patient 3 days before birth.	2 CS, 1 VD 38 + 4–40 weeks	Only performed on one sample: RT-PCR on neonatal oropharyngeal swab RT-PCR on placental samples, cord blood, plasma serum and whole blood.	Cord blood tested negative, the results of placenta samples not mentioned.
Lokken EM et al., 2020	46 (only 1 examined)	No evidence of pregnancy complications apart from marginal cord insertion. Stillbirth.	Yes, tested positive on day of admission.	VD 38 + 4 weeks*	Culture of chorioamniotic membranes, microarray of fetus, histopathology, PCR of placental parenchyma and fetal nasopharynx, fetal autopsy.	SGA placenta, acute chorioamnionitis, severe chronic villitis, mild funisitis, no viral cytopathic changes (no viral inclusions). Placenta and fetal tissues negative for SARS-CoV-2 (but delay between fetal demise and testing), culture of membranes = normal genital flora.
Mulvey JJ et al., 2020	5	No evidence of pregnancy complications. No detailed neonatal information.	Yes, all 5 tested positive during intrapartum period.	4 VD, 1 CS 38–40 weeks	IHC for complement, spike IHC, viral RNA *in situ* hybridisation, histopathology (compared to normal VD as controls).	Placental histology: 5 x fetal vascular malperfusion, 5 x thrombosis in larger vessels, 4 x intramural fibrin deposition, 1 x vascular karyorrhexis, 1 x avascular villi, 1 x perivillous fibrin. Complement staining normal, viral protein/RNA rare, thrombosis in larger vessels (3x chorionic plate, 2x stem villi).
Nie R et al., 2020	33 (5 pregnancies ongoing and one termination of pregnancy).	Three cases of preterm prelabour rupture of membranes, 2 cases of hypertensive diseases of pregnancy, 2 cases of gestational diabetes mellitus, 1 woman had spontaneous preterm labour. 11 women received antenatal corticosteroids. 10 infants born preterm. Five had birthweight <2500 g. All Apgar scores at 5 min were 9 or 10. One infant (born 34 weeks' gestation) transferred to the NICU with a diagnosis of ARDS. One neonate tested positive.	Yes, all 33 tested positive (unclear when).	5 VD, 22 CS 1 termination of pregnancy, 5 ongoing pregnancies. 18 term, 9 preterm, 1 termination of pregnancy.	RT-PCR of neonatal throat swabs (26 tests), no mention of placenta or cord blood testing.	The placenta and cord blood of the positive neonate were negative.
Patanè L et al., 2020	22 (two neonates tested positive & the cases examined).	No evidence of pregnancy complications. Normal birthweights (2660 g and 2686 g). 5 min Apgar scores 9 and 10. Case 1 neonate tested positive immediately after birth. Case 2 neonate negative after birth but positive day 7.	Yes, 22 tested positive (two of the infants tested positive, and the cases examined) (unclear when, possibly at birth).	1VD, 1 CS 37 + 1* weeks and 37 + 4weeks*	H&E, IHC and *in situ* hybridisation on placentas for spike protein mRNA. RT-PCR on neonatal swabs.	Both placentas had chronic intervillositis, with macrophages in villi and intervillous space. IHC showed CD68 macrophages. In situ hybridisation shows antigen in the syncytiotrophoblast.
Penfield CA et al., 2020	11 (out of 32 positive women).	No evidence of pregnancy complications. All live born (no other data reported). All neonates tested negative.	Yes, all tested positive. For those with positive samples: up to 2 days before delivery. For negative samples: two tested positive at delivery, remainder between 1 and 15 days before delivery.	4 CS, 7 VD 26 + 5–41 + 3 weeks.	RT-PCR on placenta, membrane and neonatal swabs.	One placental sample and two membrane samples tested negative.
Peng Z et al., 2020	1	No evidence of pregnancy complications Single course of dexamethasone for fetal lung development. Normal birthweight (2600 g). Apgar score of 10.	Yes, tested positive one day before birth.	CS 35 + 3 weeks.	RT-PCR of amniotic fluid, placenta, cord blood. Gross histology of placenta, neonatal swabs.	The placenta looked normal, all samples tested negative for nucleic acids.
Pereira A et al., 2020	60 (23 delivered, 6 placentas tested)	3 cases of pre-eclampsia, 2 premature deliveries, 5 CS (1 for maternal respiratory failure, breech position, 2 for non-progression of labour, 1 induction failure, 1 for HELLP syndrome). Two NICU admissions, 3 with FGR. 23 newborns tested negative.	All 23 tested positive, unclear when.	18 VD, 5 CS 21 term births, two preterm.	Neonates tested for SARS-CoV-2 by RT-PCR nasopharyngeal swabs, placentas tested by RT-PCR.	All six placentas tested negative.
Prabhu M et al., 2020	70	All the infants who were tested were negative. Preterm labour or preterm premature rupture of membranes: positive mothers: 3 (1.4%), negative mothers: 25 (4.1%). Chronic hypertension positive mothers: 3 (1.4%), negative mothers: 13 (2.1%). Pre-eclampsia or gestational hypertension positive mothers: 11 (15.7%) negative mothers: 56 (9.3%). Pregestational diabetes positive mothers: 4 (5.7%) negative mothers: (1.2%). Gestational diabetes positive mothers: 6 (8.6%) negative mothers: 54 (8.9%). Preterm birth positive mothers: 11 (15.7%) negative mothers: 57 (9.4%). One case admitted to ICU for hypoxia (delivered via NVD 3 days later). Multiple gestations positive mothers: 4 (5.7%) negative mothers: 15 (2.5%).	Yes, all cases tested positive at admission.	38 VD, 32 CS Median 38 + 4 weeks* (IQR: 37+2–39 + 6)*	Placental histology compared to 605 SARS-CoV-2 negative women. Placental histology: 29 placentas compared to 106 placentas from SARS-CoV-2 negative women.	FVM noted in 14 cases (48.3%) from cases, and 12 from controls (11.3%, p < 0.001). These has thrombi in the fetal vessels. MVM in 8 cases (27.6%) and 33 controls (31.1%, NS). Evidence of chorioamnionitis maternal and fetal response in 2 cases (6.9%) and 7 from controls (6.6%, NS). Chronic villitis (low grade) in 2 cases (6.9%) and 9 controls (8.5%). High grade in 3 cases (10.3%) and 4 controls (3.8%), both not significant. Umbilical cord abnormalities in 1 case (3.4%) and 17 controls (16% NS). Chorangiosis in 0 cases (0%) and 1 control (0.9%, NS). Other placental abnormalities 3 cases (10.3%) and 10 controls (9.4%, NS). One infant from SARS-CoV-2 positive mother was stillborn at 37 weeks (poorly controlled type 2 diabetes, normal placental pathology, no autopsy).
Pulinx B et al., 2020	1	Twin pregnancy. Gestational diabetes, one intrauterine death, one prepartal death.	Yes, tested positive 2 weeks before birth.	VD 24 weeks	RT-PCR on both placentas, amniotic fluid sample and amniotic sac IHC for macrophages, T cells and SARS-CoV-2. Morphology by three pathologists.	Both placentas tested negative and the amniotic fluid tested positive (tested as whole as VD). Amniotic sacs tested negative. Both placentas had extensive intervillous fibrin deposition, ischemic necrosis of surrounding villi, aggregates of histiocytes and T cells in the intervillous space (diagnosis of chronic intervillositis). Viral localisation in syncytiotrophoblast.
Richtmann R et al., 2020	5	Two cases were obese, three cases were overweight. All infants were stillborn.	Yes, tested positive between 22 days before birth and at admission.	3 VD, 2 CS 21+1–38 + 3 weeks	Histopathologist blinded to SARS-CoV-2 status. Histopathology on placenta and amniotic fluid tested for SARS-CoV-2	Case 1: Placental fragments tested positive. Pathology: acute chorioamnionitis, extensive perivillous fibrin deposition and acute villitis, findings suggestive of villitis of unknown etiology. No fetal autopsy. Case 2: no fetal malformations, fetal and placental histology showed signs of acute infection: extensive perivillous fibrin deposition, acute villitis and intervillositis. focal acute chorioamnionitis. Findings suggestive of ischemia and villitis. Case 3: placenta tested negative, amniotic fluid inconclusive. Pathology: acute chorioamnionitis, subchorionic thrombosis, findings suggestive of ischemia and infection. No fetal autopsy. Case 4: placenta and amniotic fluid not tested. Acute chorioamnionitis, acute deciduitis, findings suggestive of ischemia and infection. No fetal autopsy. Case 5: Placenta and amniotic fluid tested positive. Acute chorioamnionitis. Acute deciduitis. No fetal autopsy.
Semeshkin AA et al., 2020	20	One set of twins. No maternal information provided. All neonatal Apgar scores 8–10. All neonates tested negative via nasopharyngeal swab.	Yes, all on admission.	16 VD, 4 CS Gestation not provided	IgM and IgG antibodies in maternal and neonatal serum on days 1 or 2 after birth. Neonatal nasal and nasopharyngeal swab on day 1 or 2 after birth	Antibodies detected in all samples. IgM and IgG were raised in one case. IgM and IgG were in range for 4 neonates (levels similar to mothers). IgG was raised 14 neonates, their mothers had high titres of IgG and IgM outside of reference range. All neonates had IgG antibodies in their serum. IgG was elevated in 16 neonates, but only in cases where maternal levels were also elevated.
Shanes ED et al., 2020	16	One case of gestational diabetes and obstetric cholestasis, one with hypertensive disorders. One 16- week fetal death with preterm prelabour rupture of membranes, one preterm birth at 34 weeks' gestation. All neonates were negative including IUFD.	All tested positive; 6 x on day of birth, 2 × 1 day, 1 × 2 days, 1 × 7 days, 1 × 8 days, 2 × 25 days, 1 × 28 days, 1 x 34 days. IUFD on date of birth.	15 not specified, 1 IUFD 14 term births, one 34 weeks, one 16- week IUFD.	Placental histology compared to historical normal controls, also used controls with history of melanoma, neonatal nasopharyngeal swabs tested by PCR.	IUFD placenta: retroplacental haematoma, villous oedema, villous maturation was appropriate. No acute or chronic inflammation. 15 cases compared to controls:: Total: 11 some features of MVM, 12 with some features of FVM 11 x maternal vascular malperfusion, 1 x central infarct, 3 x peripheral villous infarct, 3 x villous agglutination, 3 x atherosis and fibrinoid necrosis of maternal vessels, 5 x hypertrophy of membrane arterioles, 2 x accelerated villous maturation, 12 x fetal vascular malperfusion, 4 x clustered avascular villi, 1 x fibrin deposition in fetal vessels, 4 x delayed villous maturation, 3 x hypercoiled cord, 4 x choroangiosis, 1 x acute inflammatory pathology, 2 x chronic inflammatory pathology, 3 x perivillous fibrin deposition.
Sisman J et al., 2020	1	Type 2 diabetes, obesity, late latent syphilis, premature preterm rupture of membranes. Normal birthweight, Apgar scores 7–9, went to NICU for monitoring (IVIG for suspected ABO incompatibility). Neonate tested positive at 24, 48 h and 14 days after birth.	Tested positive 3 days before birth.	VD 34 weeks	Neonatal nasopharyngeal swab at 24 and 48 h, placental histopathology, IHC for CD68 and SARS-CoV-2. TEM	Placental histology: patchy histiocytic intervillositis and villous associated with villous karyorrhexis and necrosis, focal basal chronic villitis, focal parabasal infarct and features of meconium exposure in the fetal membranes. SARS-CoV-2 nucleocapsid in syncytiotrophoblast. TEM: 89–129 nm structures clustered within “membrane bound cisternal spaces” in the syncytiotrophoblast.
Smithgall MC et al., 2020	51	Positive mothers: 21 women had comorbidities (41.2%), negative mothers: 12 (48%, NS). Apgar scores >7. All neonates tested negative.	Yes, tested on admission.	26 VD, 25 CS 10 born <37 weeks, 41 born > 37 weeks.	Compared to 25 SARS-CoV-2 negative women. Neonates to positive mothers tested by nasopharyngeal swab. Placentas grossly examined, umbilical cord, membranes and placenta were examined. *In situ* hybridisation or IHC for placentas from positive women.	32 placentas tested by *in situ* hybridisation and IHC, 5 by *in situ* hybridisation and 14 with IHC. No viral cytopathic changes, all negative. Placental pathology: Ascending uterine infection maternal response cases: 33.3%, 36% controls. Fetal response cases: 17.7%, controls: 12% (NS). MVM: any mention of MVM in 58 placentas (cases) and 17 placentas (controls) NS. FVM total: any mention of FVM 17 (cases) and 2 (controls) (NS). Significantly more villous agglutination in cases vs controls (41.2% vs 8%, p = 0.003) and significantly more subchorionic thrombus (17% vs 0%, p = 0.026). Chronic villitis of unknown etiology in 2 cases and 2 controls (NS).
Vivanti AJ et al., 2020	1	No evidence of pregnancy complications. Neonatal Apgar scores 4, 2 and 7. Intubated on NICU. Birthweight 2540 g. Neonate tested positive in all samples.	Yes, on admission (3 days before birth)	CS 35 + 5 weeks	Neonatal blood and bronchoalveolar lavage, nasopharyngeal and rectal swabs at 1 h of life, repeated at day 3 and 19. RT-PCR on placenta and amniotic fluid. IHC of placenta for SARS-CoV-2. Placental histology.	Amniotic fluid tested negative. Placental tested positive (highest viral load of any of the samples). Placental pathology: diffuse perivillous fibrin deposition, infarction, acute and chronic intervillositis (CD68 macrophages). Cytoplasm of trophoblast positive for N protein by IHC.
Wang S et al., 2020	1	No evidence of pregnancy complications. Methylprednisolone postnatally. Live male infant, normal birthweight (3205 g). Apgar score of 9 at 5 min. Fetal throat swab positive.	Yes. Tested positive same day after birth.	CS 40 + 1 weeks (calculated)	PCR on neonatal swabs, cord blood, amniotic fluid placental samples.	Cord blood, placental samples tested negative.
Wang X et al., 2020	1	No evidence of pregnancy complications. Dexamethasone and magnesium sulphate as fetal prophylaxis. Live male infant, normal birthweight (1870g). Apgar score of 10 at 5 min. Neonate tested negative.	Yes. Tested positive 3 days before birth.	CS 30 weeks	RT-PCR of amniotic fluid, cord blood, placenta, throat swab of neonate.	Placenta, amniotic fluid, cord blood all tested negative
Xiong X et al., 2020	1	No evidence of pregnancy complications Corticosteroid therapies given. Live infant, normal birthweight (3070 g). Five minute Apgar score of 10. Neonate was negative.	Yes, tested positive 5 weeks 2 days before birth.	VD 38 + 4 weeks	Maternal throat swab tested for COVID though RT-PCR. Neonatal IgM and IgG, PCR of amniotic fluid and neonatal throat and rectal swabs, Protein N in placenta, pathological analysis of placenta.	Amniotic fluid was negative, Placenta was negative for N protein of SARS-CoV-2 and there was no inflammation, neonatal IgM and IgG were negative.
Yu N et al., 2020	7	Increased fetal movement in one pregnancy. 6 pregnancies had no pregnancy complications 5/7 patients given methylprednisolone postnatally. All infants live born with normal birthweight (3000–3500 g). All 5 min Apgar scores 9–10. One neonate tested positive after 36 h.	Yes, all 7 tested positive; 4 x on day of birth, 1 × 1 day, 1 × 2 days, 2 x 3 days.	7 CS 37-41 + 5 weeks	Maternal Covid infection Dx with throat swap though RT-PCR. Nucleic acid test on neonate (x3) and placenta and cord blood (unclear how many samples).	Placenta and cord blood tested negative for the positive neonate.
Yin M-Z et al., 2020	31 (17 births, 3 termination of pregnancies)	One pregnancy with hypertension, one with diabetes, one cardiovascular disease. 35% of the 31 women received glucocorticoid therapies. One low birth weight (<2500 g) in premature birth. Apgar score between 7 and 8 at 1 min, between 8 and 9 at 5 min. All 17 neonates tested negative.	All 17 tested positive, unclear when.	13 CS, 4 VD 35–41 weeks	RT-PCR on amniotic fluid (x2), placenta (x2), 17 neonatal throat swabs. Maternal throat swabs.	All samples tested negative.
Zeng H et al., 2020	6	No evidence of pregnancy complications. All infants live born with 5 min Apgar scores 9–10. All neonates tested negative.	Yes, unclear when.	6 CS All third trimester	RT-PCR maternal and neonatal throat swabs and blood samples. Cytokine profiling, and IgM and IgG analysis of neonatal serum. Also, IgG and IgM for maternal serum.	Four mothers had high serum IgG and IgM. One mother with only raised IgG. All 6 neonates had virus-specific antibodies in their serum. Two samples had high IgG and IgM, three neonates had high IgG but normal IgM. IL-6 increased in all neonates.

### 3.1 COVID-19

Forty-two articles regarding COVID-19 met inclusion criteria. When there was sufficient detail to determine duplication of case reports, we included each case only once ([[14], [15], [16]], there was insufficient data to confirm or refute whether the case was also included in Ref. [17]).

Of the articles that were included, 5 were non-peer-reviewed preprints [[18], [19], [20], [21]], and 27 were case studies with 5 or fewer women (19 were single case reports). Additionally, some studies examined a small proportion of placentas from all the COVID-19 positive women who had given birth [20,[22], [23], [24], [25]] and some had ongoing pregnancies [19,21,26].

Only four placental studies used controls for comparison: Mulvey et al. used five normal placentas as controls which were assessed in the same way as the cases [27]. Prabhu et al. studied 605 women who tested negative for SARS-CoV-2 and compared the placental pathology of 106 placentas from 99 of these pregnancies [25]. Shanes et al. used two control groups; the first historical controls, the second, a group being examined due to maternal history of melanoma [28] and Smithgall et al. used 25 SARS-CoV-2 negative women as controls [29]. Only Mulvey et al. and Richtmann et al. reported that samples were blinded for assessment [27,30]. In contrast, Shanes et al. noted that the pathologist was aware of the COVID-19 infection status [28].

In total, the studies reported findings from 325 women whose pregnancy outcome was known following SARS-CoV-2 infection, seven of which were twin pregnancies [25,[31], [32], [33], [34], [35]], six pregnancies were terminated (one for maternal health reasons [18], one for personal reasons [19], three for “personal choice” [21] and one where the reason was unclear [17]). Of the remaining pregnancies, 83 births (58%) were by Caesarean section and no information was provided in 16 cases. There were 11 reports of fetal death in utero (16, 19, 21, 23, 24 (twin pregnancy), 28, 30, 37 and two at 38 weeks’ gestation) [22,25,28,30,34,36].

#### 3.1.1 Maternal clinical data

Eighteen studies, involving 45 pregnancies, reported no evidence of pregnancy complications in their cases [16,22,23,26,27,[37], [38], [39], [40], [41], [42], [43], [44], [45], [46], [47], [48], [49]]. There was no apparent increase in the frequency of maternal pregnancy complications in the remaining cases (see Table 2).

**Table 2:** **Maternal medical complications during pregnancy following SARS-CoV-2 infection.** Proportion of women presenting with medical conditions and as a percentage of total cases. # cases only included if specified as spontaneous preterm birth.

Maternal Medical Condition	Number of women	Percentage of total cases examined (%)	References
Gestational diabetes	7	2	[19,20,28,33,60]
Pre-existing diabetes	2	0.6	[21,57]
Hypertensive disorders of pregnancy	18	5	[18,19,21,24,25,28,31,53,60]
Obstetric cholestasis	1	0.3	[28]
Acute cholecystitis	1	0.3	[52]
Cardiovascular disease	1	0.3	[21]
Metabolic acidosis	1	0.3	[51]
Respiratory failure	2	0.6	[24,25]
Preterm prelabour rupture of membrane	9	2.5	[19,25,33,57]
Spontaneous preterm birth^#^	6	1.7	[19,31,33,34,36,57]
Placenta previa	3	0.8	[31,52]
Placental abruption	3	0.8	[18,32,52]
Systemic lupus erythematosus (SLE)	1	0.3	[42]

The timing of infection relative to delivery varied widely; 186 women tested positive for SARS-CoV-2 on the day of delivery, 27 tested positive up to a week before birth, 6 tested positive up to two weeks before [26,28,30,34,50], 8 tested positive up to a month before [17,26,28,30,45] and 2 tested positive up to 3 months before [28,39]. Four women were admitted for delivery, but it is unclear when they were diagnosed [31,51]. Seven women were diagnosed postnatally [33,37,42,52]; although one woman's swab tested negative, the CT scan indicated that she had COVID-19 [52]. The timing of diagnosis was unclear for 85 women.

Corticosteroids were administered to a total of 43 women (in 15 reports) [16,17,19,20,32,33,[37], [38], [39],^41^,^45^,^46^,^49^,^51^,^53^]. Four studies reported that the corticosteroids were primarily given for prophylaxis against fetal lung disease [32,38,49,51]. Yin et al. noted that 11 of 31 women had glucocorticoid therapy, but it is unclear which of the women had given birth [21]. The remaining studies did not state whether any corticosteroids were administered to the mothers during their hospital admission for COVID-19.

#### 3.1.2 Neonatal outcomes

A total of 167 infants were born after 37 weeks' gestation, and 58 were born preterm (including four sets of twins), with the majority being born after 30 weeks' gestation. Seven infants were born in the “third trimester” (not defined by studies) and the exact gestation of 95 infants was unclear [17,25,35], with one study providing median gestational ages and the interquartile range which included four multiple-pregnancies [25]. There were six terminations of pregnancy including one at 22 weeks' gestation [18]. In another case the outcome of the pregnancy was unclear [17] Two infants were described as small-for-gestational-age (SGA), but no definition was provided [31], three infants had FGR or SGA, but no definitions were provided [24] and 11 birthweights were recorded as <2500 g, with no comment on whether the weights were appropriate for gestational age [19,21,32,33]. The birthweight of 5 stillborn infants were provided: 1070 g, 329 g, 2895 g, 680 g, and 1430 g at 28, 21, 38, 23 and 30 weeks’ gestation, respectively [30].

#### 3.1.3 Placental histopathological findings following SARS-CoV-2 infection

##### 3.1.3.1 Third trimester

Twenty studies reported histological or histopathological findings of the examination of 150 third trimester placentas from women with SARS-CoV-2 infection.

**Fig. 2:**
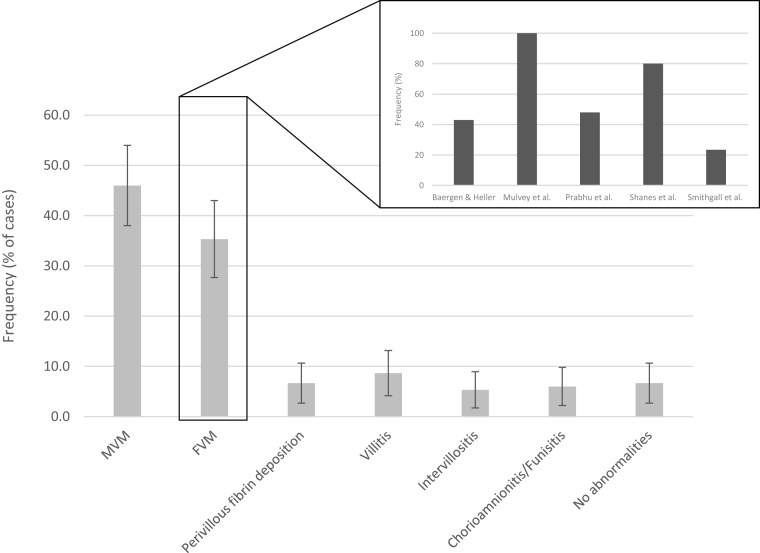
**Proportion of third trimester cases with placental histopathological lesions or showing no abnormalities of placental pathology in women with SARS-CoV-2 infection.** Error bars show 95% confidence intervals. Inset shows that estimates of FVM by individual studies vary in frequency from 23.5 to 100%. MVM: maternal vascular malperfusion, FVM: fetal vascular malperfusion.

Hypoxia induced by maternal SARS-CoV-2 infection may lead to maternal vascular malperfusion (MVM) [54]; features or diagnoses of MVM were noted in 69 cases (46% of placentas examined, 95% confidence interval (CI) 38.1–53.9%) by 12 studies [25,[28], [29], [30], [31], [32],^34^,^43^,^44^,^51^,^52^,^55^] (Fig. 2). Shanes et al. found some evidence of MVM in 11/15 cases (72%), with a formal diagnosis in 2 cases [28]. The number of cases with any features of MVM was significantly increased compared to both control groups (melanoma controls p = 0.001 and historical controls p = 0.046). In contrast, Baergen and Heller reported that 3/21 placentas (14%) had features of MVM [31]. Smithgall et al. reported 38/51 placentas had evidence of MVM, compared to 16/25 healthy controls (not-significant), but the frequency of villous agglutination and subchorionic thombi were significantly increased in the placentas of SARS-CoV-2 positive mothers (p = 0.003 and 0.026, respectively) [29]. Prabhu et al. found 8/29 placentas had evidence of MVM compared to 33/106 healthy controls (not- significant) [25].

Fetal vascular malperfusion (FVM) is associated with a lack of blood flow within the placenta and is significantly associated with reduced placental weight, stillbirth and FGR [56]. Features or diagnoses of FVM were noted in 53 cases (35.3% of placentas examined, 95% CI 27.7–42.9%) by 6 studies [25,[27], [28], [29],^31^,^57^] (Fig. 2). Shanes et al. found 12/15 cases (80%) with some features of FVM, but only one had a formal diagnosis [28]. Mulvey et al. reported all five of the placentas examined had FVM, which was absent in controls [27]. Nine out of twenty-one (43%) placentas examined had evidence of FVM in the study by Baergen and Heller [31] (Fig. 2 inset). Smithgall et al. found 12/51 placentas had evidence of FMV compared to 2/25 controls (non-significant) [29], whilst Prabhu et al. reported significantly more placentas (14/29) had evidence of FVM compared to 12/106 controls (p < 0.001) [25].

Six studies reported perivillous fibrin deposition in 10 placentas [27,28,30,31,44,52]. Chen S et al. reported various degrees of fibrin deposition inside and around the villi [52]. Increased “syncytial nodules”, more commonly described as syncytial nuclear aggregates (SNAs), were noted in two of the placentas [52].

Inflammatory changes in the placenta can be a result of maternal and fetal inflammatory response often to infective microorganisms, such as chorioamnionitis [58]. Inflammatory changes were reported in 10 studies. In one study, both cases born at term had CD68^+^ macrophages in the villi and intervillous space and were diagnosed as having chronic intervillositis [23]. High grade chronic villitis was detected in one of the placentas of a twin pregnancy, with an additional case of high grade chronic villitis and associated avascular villi and two cases of chronic villitis were also reported [31]. Villitis was reported in five placentas [29,43,44,57], whilst histiocytic villitis was found in two placentas [55,57]. In one study, chorioamnionitis was reported in placentas from three stillborn infants, with one also having the finding of villitis [30]. Additionally, the placentas of a 24 week twin pregnancy where both infants were stillborn both had the findings of chronic intervillositis [34]. Baergen and Heller also reported a case with ascending infection with acute chorioamnionitis and funisitis, one case of early acute funisitis, and six cases of “meconium macrophages” [31]. Four cases of chorangiosis, one case with “acute inflammatory pathology” and one case of “chronic inflammatory pathology” were reported [28]. The number of cases with “acute inflammatory pathology” was significantly lower compared to both control groups (melanoma cohort p = 0.001 and historical cohort <0.001), whereas the number of cases with “chronic inflammatory pathology” did not differ. A 38-week stillbirth placenta had evidence of acute chorioamnionitis, severe chronic villitis, mild funisitis but no viral cytopathic changes [22]. However, the exact time of fetal demise was unknown and there would have been a delay before tissue sampling could occur, which may account for the findings.

Other histopathological findings have been reported in pregnancies with adverse outcomes, such as umbilical cord coiling in the placentas of SGA infants and following stillbirth [59]. Three further studies reported information on the umbilical cord: one furcate insertion [31], one case of a marginal insertion with a hypercoiled umbilical cord [31], one case of a furcate insertion with a long umbilical cord [27] and three cases with a hypercoiled umbilical cord [28]. The umbilical cord vessels were reported to be hyperspiralized in one placenta, along with the finding of delayed villous maturation and “capillary congestion” in terminal villi [42]. Delayed villous maturation was noted in four cases (27%) by Shanes et al.; a significant increase compared to both the melanoma controls and historical controls (p = 0.006 and < 0.001, respectively) [28]. Deciduitis was reported in one stillbirth case [30] and decidual vasculopathy was noted in one case in a 28-week pregnancy with focal villous oedema in mature chorionic villi [51], one placenta from a twin pregnancy had a villous infarct [31] and a chorionic haemangioma was found in one placenta [52]. Three studies reported placental abruption [18,32,52]. In contrast, several studies reported no abnormalities of the placenta [17,33,49,60] and umbilical cord [31]. Kuhrt et al. and Xiong et al. reported no evidence of inflammation in placentas of a twin pregnancy and normal 38 week pregnancy respectively, but neither commented whether the placental was histologically normal [32,39]. The placenta of a 37 week stillborn infant was reported as being histologically normal [25].

Patanè et al. reported strong staining of the SARS-CoV-2 spike protein mRNA in the syncytiotrophoblast by *in situ* hybridisation [23], conversely Mulvey et al. reported that evidence of the viral spike protein was rare [27]. The same authors also compared complement staining and found that the level of staining was similar to controls. Hsu et al. reported the finding of the SARS-CoV-2 antigen throughout the placenta, including in endothelial cells, but rarely in the syncytium [43], whilst two studies identified SARS-CoV-2 in the syncytiotrophoblast, including two placentas from stillborn twins [34,57]. Vivanti et al. identified the SARS-Cov-2 N protein in the cytoplasm of trophoblast [44]. Finally, Xiong et al. reported negative findings of Protein N in one placenta [39] and Smithgall et al. found no evidence of SARS-CoV-2 in 51 placentas by both immunohistochemistry and *in situ* hybridization [29].

##### 3.1.3.2 First and second trimester

This review identified six studies where terminations or miscarriages occurred. In two cases of termination of pregnancy, there was no placental examination or testing for the presence of SARS-CoV-2 in the placenta or fetus [17,19]. One placenta from a 16-week fetal demise was identified as having a retroplacental haematoma and villous oedema [28]. However, the villous maturation was deemed appropriate and there was no evidence of acute or chronic inflammation. In contrast, the remaining pregnancy losses showed evidence of inflammation and fibrin deposition, with one case (19-week miscarriage) demonstrating monocytes and neutrophils in the subchorial space along with increased intervillous fibrin deposition and funisitis [36]. A 22-week miscarriage had diffuse perivillous fibrin and a focal placental infarct along with the presence of both macrophages and T cells in placental tissue [18]. In a 21-week stillbirth, histopathological examination described villitis, intervillositis and extensive perivillous fibrin deposition and a 23 week stillbirth had acute chorioamnioinitis and deciduitis [30]. As chorioamnionitis and funisitis are usually associated with a bacterial or fungal infection, these observations may reflect a secondary infection, rather than a primary response to SARS-CoV2 infection.

#### 3.1.4 Ultrastructural analysis

Two studies reported findings using transmission electron microscopy. Hosier et al. reported particles between 75 and 100 μM, identified as SARS-CoV-2 viral particles by the authors, in the “cytosol of placental cells” [18] in a placenta was from a pregnancy terminated at 22 weeks’ gestation due to severe preeclampsia. The sample was taken from near the umbilical cord insertion point with the ultrastructure of placenta reported as being “well-preserved”. Algarroba et al. reported virions in the syncytiotrophoblast, microvilli and in cells thought to be fibroblasts in terminal villi [51] in a placenta from a 28-week delivery due to maternal acidosis. Finally, Sisman et al. reported virion-sized structures located within “membrane-bound cisternal spaces” in the syncytiotrophoblast [57]. To date, no placental ultrastructural studies have demonstrated specific labelling of SARS-CoV-2 (e.g. by immunogold), which would represent more definitive evidence of viral infection of any placental cells.

#### 3.1.5 Evidence of SARS-CoV-2 infection in neonates/placentas

Four studies tested fetal tissues following terminations, miscarriages or stillbirths reported negative PCR results for SARS-CoV-2 [18,22,28,36]. Baud et al. tested the placental cotyledon and placenta “submembrane”, both of which were positive for SARS-CoV-2 RNA, but the amniotic fluid was negative [36]. Hosier et al. reported a positive result for SARS-CoV-2 RNA in the umbilical cord and the placental samples [18]. These authors also reported positive identification of the virus in the syncytiotrophoblast via *in situ* hybridisation. Lokken et al. reported negative PCR results in the placenta following stillbirth, although the time interval between fetal demise and sampling may have adversely affected sample quality [22]. The placentas of four stillborn infants, including a set of DCDA twins, tested positive for SARS-CoV-2 [30,34], whilst one placenta tested negative [30]. The amniotic fluid of the twin pregnancy tested positive, whilst the amniotic sacs tested negative [34].

Of the studies reporting live births, 34 tested some or all the neonates for SARS-CoV-2. Out of 307 neonates tested, 7 (2%) were positive: three within 24 h of birth [23,55,57], two within 36 h of birth [37,41] (although these studies may report the same infant), one 7 days after delivery [23] and for one infant it was unclear when testing for SARS-CoV-2 was carried out [19].

Across all studies, the following samples were tested for the presence of SARS-CoV-2 RNA: placenta (19 studies), placental membranes (2 studies), amniotic fluid (10 studies), umbilical cord (3 studies), and umbilical cord blood (11 studies). Of the 19 studies that tested for SARS-CoV-2 RNA in the placenta [16,17,[19], [20], [21],^24^,^26^,^37^,^38^,^41^,^42^,^44^,[46], [47], [48], [49],^52^,^53^,^55^], 14 reported negative results (three neonates tested positive [19,26,41]), 4 studies reported positive results [26,42,44,55] and one study did not report the findings [20]. Additionally, 2 studies reported testing placental membranes with 2 positive [26], and 3 negative results [52]. One study reported a negative umbilical cord result for SARS-CoV-2 RNA [52]. Ten studies reported negative results of testing for SARS-CoV-2 in amniotic fluid [16,17,21,[37], [38], [39],^44^,^46^,^48^,^49^] and 11 in umbilical cord blood [16,17,19,20,37,38,41,[46], [47], [48], [49]].

Five studies tested for the presence of IgM and IgG antibodies. Dong et al. reported one case of a neonate born at 37 week's gestation who tested positive for both IgM and IgG antibodies shortly after birth [45]. However, the neonatal swabs tested negative for SARS-CoV-2. Zheng H et al. reported the antibody results of six neonates born during the third trimester [40]. Despite throat swabs testing negative, two infants had IgM, and all six had IgG, although one was not above the level of the reference range. Conversely, one infant born at 38 weeks' gestation tested negative for both IgM and IgG antibodies, with the mother testing positive five weeks before delivery [39] and one infant tested negative for both IgM and IgG 10 days after birth [42]. Semeshkin et al. reported the IgM and IgG results of 21 neonates, tested 1 or 2 days after birth; one case had raised levels of both IgM and IgG and only IgG was raised in 14 neonates [35].

### 3.2 SARS-CoV and MERS-CoV

In total eight articles met the inclusion criteria. Characteristics of included studies are described in Table 3. Of the papers investigating SARS-CoV-1, five were case studies with five or fewer women (four were single case reports [[61], [62], [63], [64]]). One study examined the placentas from five out of twelve women included in the larger study [65]. It is possible that the studies by Wong et al., Shek et al. and Ng et al. report the same cases [50,65,66].

**Table 3:** **Characteristics of SARS-CoV-1 and MERS-CoV included studies.** Calculated: when gestation at birth was calculated according to timeline of events, VD: vaginal delivery, CS: Caesarean section, PCR: polymerase chain reaction, IHC: immunohistochemistry.

Study	Number of women	Clinical data	Mothers SARS/MERS positive	Method of delivery and gestational age at birth	Investigations	Results
Jeong SY et al., 2017 MERS-CoV	1	Placental abruption. Normal birthweight (3140 g). Apgar score 9 at 5 min. Neonate tested negative for MERS-CoV.	Yes, tested positive 16 days before delivery.	CS 37 + 5 weeks	PCR on placenta and cord blood, antibody detection in neonate. RT-PCR on nasopharyngeal swab and peripheral blood from newborn.	Placenta and cord blood tested negative. Placental abruption. No MERS-CoV antibodies IgM, IgG or IgA were found in the neonate.
Jiang X et al., 2004 SARS-CoV-1	1	No evidence of pregnancy complications. Suspected SARS prompted termination of pregnancy.	Yes, tested positive, 17 days before termination.	Termination of pregnancy 8 months (calculated)	SARS-IgG detection kit, indirect immunofluorescence, ELISA on serum/plasma, Western blot on nucleocapsid “N" protein on maternal blood, cord blood, amniotic fluid, PCR on maternal blood, cord blood and amniotic fluid.	Western blot showed n protein in IgG in maternal blood, cord blood and amniotic fluid. IFA also positive. No SARS-CoV genes detected in maternal blood, umbilical blood, or fluid using a SARS virus fluorescence qPCR.
Ng WF et al., 2006 SARS-CoV-1	7	No evidence of pregnancy complications, but two cases with oligohydramnios. Two maternal deaths. Methylprednisolone given to 4 patients. Four AGA. One pregnancy electively terminated. Two infants had suspected fetal growth restriction; both were small for gestational age. All term infants had Apgar score of 9 or 10 at 5 min. All the neonates were negative for SARS-CoV-1 after “investigations"	Yes, tested positive 1 x same week as delivery, 1 × 1 week before, 1 × 5 weeks before, 1 × 7 weeks before, 1 × 35 weeks before delivery. Termination of pregnancy = 3 weeks before.	4 CS, 2 VD, 1 termination of pregnancy Termination of pregnancy at 15 weeks, 26–38 weeks	Placental pathology. “Investigations” for SARS-CoV-1 infection.	P1: gestation = 28 weeks, prominent intervillous fibrin, maternal death. P2: gestation = 32 weeks prominent intervillous fibrin and increased calcification, maternal death. P3: gestation = 26 weeks, focal subchorionic fibrin. P4: gestation = 33 weeks, accelerated villous maturation and avascular villi (19%), stem villi vessel obliteration, hyalinised stroma, calcification, haemosiderin granules, accelerated villous maturity with increased SNA and capillarisation. P5: gestation = 37 weeks, avascular villi (8%), infarcts (9%), increased calcification. P6: Gestation at termination: 15 weeks, increased calcification. P7: gestation: 38 weeks, normal placenta. P4+5 lesions account for around 50% of placenta. Erythroblasts found in three placentas. Two had low placental weight.
Robertson CA et al., 2004 SARS-CoV-1	1	Gestational diabetes, placenta previa. Normal birthweight (3145 g). 5 min Apgar score 9.	Yes, tested positive 26 weeks before delivery.	CS 38 weeks	RT-PCR on placenta, cord blood and amniotic fluid. IHC staining on placenta for SARS. Cord blood tested for antibodies.	Cord blood tested positive for antibodies. Placenta, cord blood and amniotic fluid tested negative. Placenta was negative for IHC SARS. Gross and microscopic inspection of the placenta did not show major abnormalities.
Shek CC et al., 2003 SARS-CoV-1	5	No evidence of pregnancy complications. Four cases had hydrocortisone. Three cases had methylprednisolone. All neonates tested negative. One infant small for gestational age. Infants had Apgar scores of 7–10 at 5 min.	Yes, all 5 tested positive; 3 x between 6 and 7 days of fever onset,1 × 6 weeks, 1 x 7 weeks.	Not specified 26–37 weeks	RT-PCR on placental swab. Tested neonatal samples.	All samples tested negative.
Stockman et al., 2004 SARS-CoV-1	1	Elevated blood glucose, early spontaneous rupture of membranes, fetal distress. Fetal distress, Apgar: 7 at 1 min, 8 at 5 min. Neonate tested negative.	Yes, tested positive 23 weeks before delivery.	CS 36 weeks	Tested neonatal nasopharyngeal swab by PCR. Tested placenta and cord blood for antibodies.	All samples tested negative.
Wong SF et al., 2004 SARS-CoV-1	12 (only 5 placentas examined).	No evidence of pregnancy complications, but 2 maternal deaths (in those who contracted disease in late pregnancy). Two patients had oligohydramnios. 4/7 diagnosed in 1st trimester miscarried and 2 had termination of pregnancies, one 1st trimester diagnosis successful ongoing pregnancy. Hydrocortisone (10/12 patients), methylprednisolone (9/12) and oral prednisolone (10/12). Two SGA, 3 AGA, one spontaneous preterm birth. Neonates tested negative.	Yes, all 12 tested positive (5 successful deliveries tested positive), 2 x same week as delivery, 1 × 1 week, 1 × 5 weeks, 1 x 7 weeks	3 miscarriages, 3 pregnancies ongoing, 4 CS, one VD 26–37 weeks.	Placental histology, RT-PCR of neonatal samples, cord blood, placental tissue, and amniotic fluid.	Three placentas were normal, two FGR placentas had avascular thrombotic terminal villi with thrombotic vasculopathy in some stem villi. Placental infarction (10%) was evident in one placenta. No placentas showed chorioamnionitis, funisitis, villitis, viral inclusion, or other features of infection. No significant erythroblastosis was noted. All samples tested negative for nucleic acids.
Yudin MH et al., 2005 SARS-CoV-1	1	No evidence of pregnancy complications. Methylprednisolone and prednisolone. Live female infant, normal birthweight 3086 g. Apgar score 9 at 5 min. Neonate tested negative.	Yes, tested positive, 6 weeks before delivery, (Antibodies in blood samples positive, original nasopharyngeal and throat swabs negative).	VD 39 + 2 weeks	RT-PCR on amniotic fluid, placenta, membranes, cord blood. Neonatal nasopharyngeal and throat swabs.	Umbilical cord, cord blood, fetal membrane, placenta, and amniotic fluid (aspirated from vagina) all tested negative.

#### 3.2.1 Placental histopathological findings following SARS-CoV-1 or MERS-CoV infections

Only one paper was identified as examining the placenta of a 37 week infant following maternal infection with MERS-CoV [67]. PCR was performed on the placenta and cord blood, both of which were negative. The neonate tested negative for MERS-CoV and did not test positive for IgM, IgG or IgA.

Five papers examined the placentas of women who had SARS-CoV-1 infections [50,[61], [62], [63], [64]]. Placental histopathology was conducted on seven specimens in one study, with one placenta being noted as normal [50]. Evidence of maternal (MVM) and fetal (FVM) vascular malperfusion were found in two placentas, and increased capillarisation and syncytial nuclear aggregates (SNAs) found in one of the two placentas [50]. Two placentas had prominent intervillous fibrin, and one placenta had focal subchorionic fibrin. Calcification was found in four placentas, including a 15-week termination, with one also having hyalinised stroma and haemosiderin granules. Finally, erythroblasts were identified in three placentas. Another study found no abnormalities following gross and microscopic inspection [62].

Across all studies, the following samples were tested to determine whether the SARS-CoV-1 virus was present: placenta (two studies with negative results by PCR [62,64], one study finding no antibodies [63]), amniotic fluid (two studies with negative results by PCR [62,64], one study found IgG [61]), cord blood (two studies found antibodies [61,62], one study did not [63]). One study reported no SARS-CoV-1 in umbilical cord and blood and “fetal membrane” samples [64]. Jiang et al. also reported negative PCR testing for SARS-CoV-1 in umbilical cord blood and amniotic fluid [61]. Three studies reported negative results for SARS-CoV-1 in neonates (2 by PCR [63,64] and 1 following “close monitoring and investigations” [50].

## 4 Discussion

This review included 20 studies reporting histopathologic findings of 150 third trimester placentas. A wide variety of histopathological abnormalities including MVM, FVM and signs of inflammation were reported with varying frequencies. The estimates of the frequencies of these lesions remain uncertain as the majority of studies reported small numbers of cases, did not use control groups and examination was not blinded to the clinical condition. Although, the presence of lesions was reported in placentas from women with COVID-19 infection, the severity of lesions was not compared between women with and without the disease. Most reports found no evidence of viral infection detected in placental tissue in COVID-19 or related infections.

This study is strengthened by the systematic nature of the search strategy run on multiple databases and standardised data collection. To maximise information obtained, pre-prints were included; as this information has not been peer-reviewed, the data may be less robust. This methodological approach is hampered by publication bias, which may prevent publication of negative findings, thus could result in overestimation of the frequency of placental pathology in women with COVID-19. As the SARS-CoV-2 pandemic originated less than 10 months ago, it is likely that further studies of placental tissue will be published and the evidence-base in this field will grow.

### 4.1 No evidence for vertical transmission via the placenta

There is limited evidence in the literature to support the possibility of transplacental transmission. Entry of SARS-CoV-2 into host cells is mediated by the viral spike (S) protein which relies on binding to a corresponding receptor on the surface of the target cell [68]; the SARS-CoV-2 cellular receptor is thought to be angiotensin-converting enzyme 2 (ACE2) [68,69]. A recent single cell transcriptome study of placental tissues and chorioamniotic membranes revealed negligible co-expression of the ACE2 and the serine protease, TMPRSS2, which mediate viral entry [70]. Alternatively, Celik et al. propose that the lack of vertical transmission is due to the absence of caveolin in syncytiotrophoblast [71,72]. Caveolae are membrane bound structures which, amongst other roles, endocytose certain viruses [73]. Celik et al., propose that the lack of caveolin results in a failure of inflammation to occur, which results in the syncytiotrophoblast remaining a continuous layer and thus SARS-CoV-2 cannot cross into the placental villi [71]. However, there may be alternative pathways used by SARS-CoV-2 to enter cells that have yet to be determined.

One study identified lesions and reported evidence of viral mRNA by *in situ* hybridisation but, critically, viral particles were only seen in the syncytiotrophoblast, rather than passing through the basement membrane into the villous stroma where it would be high-risk for the virus to enter the fetal circulation. We conclude from these studies that, while placental infection may occur rarely, this is not apparent in the majority of cases. Notably, one of the cases where viral particles were seen in the placenta, the mother had exceptionally high antibody titres [18]. In such cases, virions might be phagocytosed by the placenta as immune complexes (IgG mediated) rather than by direct invasion of the placental tissue.

Five studies reported testing neonates for the presence of IgM and IgG antibodies. IgG is the only antibody known to cross the placenta [74]*.* IgM is the first antibody produced during a microbial infection [75], and so the presence of this would appear to indicate the vertical transmission of SARS-CoV-2. However, considering the number of women that have been infected and the low number of neonates that have tested positive, vertical transmission is rare. Interpreting the impact of SARS-CoV-2 infection on the human placenta is presently constrained by available data. However, both lesions affecting the fetal and maternal vascular beds and those of inflammatory origins appear more frequently than reported in control groups and from existing studies of unselected populations; for example, Pathak et al. reported maternal vascular malperfusion and fetal thrombotic vasculopathy in 7.7% and 1% of “normal pregnancies” respectively [76]. These observations were also consistent with observations of FVM and MVM in women who had SARS-CoV-1 infection. The presence of FVM, MVM and inflammatory lesions in COVID-19 and SARS may be significant because these are seen more frequently in cases of stillbirth and pregnancy loss [77]. In particular, earlier pregnancy losses are associated with MVM or inflammation [78,79]. Presently, the association between COVID-19 and pregnancy loss is unclear, but identification of these placental histopathological lesions means an association is biologically plausible. However, placental changes could also result from periods of maternal hypoxaemia [80] observed in COVID-19, systemic inflammation or from administration of therapeutic agents such as corticosteroids, all of which are known to alter placental cell turnover and function [81].

Analysis of placental tissue in women with COVID-19 is essential to providing a mechanistic understanding of any association observed between infection with SARS-CoV-2 and adverse pregnancy outcome in epidemiological studies. Robust study designs including contemporaneous controls, blinded analyses and multiple methods to describe placental morphology and pathology will increase understanding of the nature of placental pathology seen in women with COVID-19.

Analysis of circulatory cytokines in patients with COVID-19 demonstrates an increase in levels of pro-inflammatory cytokines, with a further increase in the variety of pro-inflammatory cytokines in those most critically ill [82]. A pro-inflammatory shift in the maternal circulation has been observed in the presence of placental pathology [83], but there have yet to be any studies evaluating inflammatory markers in the maternal circulation during and after recovery from SARS-CoV-2. Measurement of cytokines and correlation to placental inflammation would provide a mechanistic link between the maternal syndrome and inflammatory placental lesions. Importantly, most placentas examined were from mothers who had COVID-19 at the time of birth or in the third trimester when there may have been insufficient time for chronic lesions to develop. Therefore, there is a need to investigate the effect that SARS-CoV-2 may have on the structure and function of placentas when infection occurs during the first or second trimester and the pregnancy continues to term, as well as on the acute effects of COVID-19.

## 5 Conclusion

Given the crucial role the placenta plays in the development and maintenance of the fetus, there is a clear need for studies on the impact of COVID-19 on pregnancy outcome and to incorporate placental tissue collection and assessment. Collaboration to maximise sample numbers analysed in individual experiments will also increase the reliability and generalisability of study findings. It is anticipated that a better understanding of the association between maternal SARS-CoV-2 infection and placental dysfunction and/or pathology will inform the delivery of maternity care during the pandemic.

## Funding information

This work was supported by the 10.13039/501100000265Medical Research Council (Grant number MR/N010892/1, MS and AH), Tommy's Charity (DH, SL, CB, AK, CJ, AH), and 10.13039/100000060National Institute of Allergy and Infectious Diseases (Grant numbers AI133976, AI145890, AI144938 and AI143265, KAW).

## Declaration of competing interest

The authors declare that they have no conflicts of interests.
