# Supplementary material for: A structured review of placental morphology and histopathological lesions associated with SARS-CoV-2 infection
Source: Placenta. 2020 Nov;101:13–29. doi: 10.1016/j.placenta.2020.08.018 (PMC7443324; doi:10.1016/j.placenta.2020.08.018)
Supplement: Multimedia component 1 [file mmc1.docx]

Medline search strategy

1. Placenta*
2. “PLACENTA”/
3. Placenta/anatomy and histology [MeSH]
4. “PREGNANCY”/
5. Pregnan*
6. 1 OR 2 OR 3 OR 4 OR 5
7. SARS
8. MERS virus
9. Coronavirus, sars [MeSH]
10. Coronavirus, sars related [MESH]
11. “SARS virus”/
12. COVID-19
13. SARS-COV-2
14. Coronavirus
15. 7 OR 8 OR 9 OR 10 OR 11 OR 12 OR 13 OR 14
16. 6 AND 15
17. Limit 16 to Humans
